# Call combination patterns in Icelandic killer whales (*Orcinus orca*)

**DOI:** 10.1038/s41598-023-48349-1

**Published:** 2023-12-08

**Authors:** Anna Selbmann, Patrick J. O. Miller, Paul J. Wensveen, Jörundur Svavarsson, Filipa I. P. Samarra

**Affiliations:** 1 Faculty of Life and Environmental Sciences, University of Iceland, Reykjavík, Iceland; 2 Sea Mammal Research Unit, School of Biology, University of St Andrews, St Andrews, UK; 3 Institute of Research Centres, University of Iceland, Vestmannaeyjar, Iceland

**Keywords:** Behavioural ecology, Animal behaviour

## Abstract

Acoustic sequences have been described in a range of species and in varying complexity. Cetaceans are known to produce complex song displays but these are generally limited to mysticetes; little is known about call combinations in odontocetes. Here we investigate call combinations produced by killer whales (*Orcinus orca*), a highly social and vocal species. Using acoustic recordings from 22 multisensor tags, we use a first order Markov model to show that transitions between call types or subtypes were significantly different from random, with repetitions and specific call combinations occurring more often than expected by chance. The mixed call combinations were composed of two or three calls and were part of three call combination clusters. Call combinations were recorded over several years, from different individuals, and several social clusters. The most common call combination cluster consisted of six call (sub-)types. Although different combinations were generated, there were clear rules regarding which were the first and last call types produced, and combinations were highly stereotyped. Two of the three call combination clusters were produced outside of feeding contexts, but their function remains unclear and further research is required to determine possible functions and whether these combinations could be behaviour- or group-specific.

## Introduction

Communication is generally thought to consist of a signal produced by a sender that is received and interpreted by one or several receivers^1^. To encode a variety of messages, an animal can increase its repertoire by creating new signals, and repertoire size is thought to be one indicator of communicative complexity^2,3^. In acoustic communication, this mechanism is limited by the abilities of the sender to produce novel sounds and of the receiver to perceive and differentiate them. This limitation can be overcome by combining sounds from a smaller vocal repertoire into a sequence, which is likely to be more efficient and less prone to errors^4^. Thus, it is not surprising that vocal sequences have been reported for a variety of species from different taxa^5^.

The simplest form of a vocal sequence is the repetition of the same signal, where either the number of repetitions or the length of the pause between signals can be of importance. For example, the number of repetitions encodes different contexts in pied babblers (*Turdoides bicolor*)^6^ and alarm calling rates increase with urgency in several mammal species^7–9^. More complex vocal sequences can be made up of two or more different signals, with the number of different sounds, their order, or timing providing information^5^. Such mixed call combinations can range from simple combinations of two sounds to complex displays such as bird or whale song with multiple distinct units. Putty-nosed monkeys (*Cercopithecus nictitans*), for example, combine two alarm calls into sequences that elicit different responses from conspecifics depending on the order in which the calls are given^10,11^. In many bird species, on the other hand, it seems that the overall diversity of sounds rather than their specific order is of importance^12^.

Cetaceans are known to produce complex song displays, but these appear to be generally limited to mysticete species, such as humpback (*Megaptera novaeangliae*) or bowhead whales (*Balaena mysticetus*)^13,14^. In the odontocetes, several species are known to produce repeated vocalisations^15–20^. The ‘social complexity hypothesis’ suggests that sociality requires high communicative complexity^2^, which should promote call combinations. While socially complex lifestyles are common in this group^21^, knowledge on mixed call combinations remains limited^22^. Bottlenose dolphins (*Tursiops truncatus*) produce non-random sequences of whistles^23,24^ and so-called bray sequences, which are related to feeding^25–28^. Stereotyped vocal sequences have also been reported for northern right whale dolphins (*Lissodelphis borealis*)^29^, striped dolphins (*Stenella coeruleoalba*)^30^, long-finned pilot whales (*Globicephala melas*)^15^, narwhals (*Monodon monoceros*)^31^, and killer whales (*Orcinus orca*)^32–35^.

Killer whales produce stereotyped calls that are thought to be important group and population markers^32,36^. In some populations, dialects have been described, with more closely related individuals sharing larger parts of their repertoire, while in other populations calls seem to be shared more widely across social groups^32,37–40^. Calls are learned, rather than innate^41,42^ and vocal repertoires are stable over long periods of time in many populations^32,43–45^. Most calls do not seem to be behaviour specific but the frequency with which they are used may vary with behavioural or social context^33,46–48^.

Early studies on killer whale acoustic signals noted that certain calls are highly repetitive, but while some reported no clear structured sequences or patterns^49^, others described calls being organised into themes, occurring in a specific order, comparable to humpback whale song^50^. In the following years, more extensive studies confirmed that killer whale calls tend to be given in repetition and that these repetitions can be given by the same individual or in exchange between individuals^18,33,34^. A few studies from the North Pacific also noted combinations of specific call types^32–34,37^, such as call types N7 and N8 in the resident killer whales. N8 never occurred without being preceded by N7, but N7 was not always followed by N8^32,33^. In Norway, so-called compound calls were described, which consist of more than one discrete call type^51,52^. It has been suggested that calls in this population are assembled from smaller subunits^53^, leading to the idea that combining calls might be a mechanism in the evolution of new signals^41^. However, considering its social complexity and how strongly social association patterns are reflected in their call repertoires, surprisingly little attention has been given to the way different call types may be combined in this species.

Here, we use data from animal-borne recording devices to investigate call combinations produced by Icelandic killer whales. We identified these sequences using strict criteria on call quality and timing, as well as a first order Markov model to quantify call transitions. Furthermore, we investigated which individuals or groups may emit call combinations and whether they are related to feeding contexts.

## Results

A total of 22 multisensor tags were deployed (21 Dtags and one CATS tag) in five years between 2009 and 2022, resulting in 112 h and 48 min of acoustic recordings (Table 1). From these recordings 8,045 high-quality calls were extracted, of which 7058 calls (87.7%) were classified into 70 distinct call categories (call types or subtypes). The remainder of calls were considered variable and not included in the analysis. Using 5624 transitions, a bout criterion interval (BCI) of 1.72 s was estimated based on maximum likelihood estimation. Only transitions between calls that fell within 1.72 s of each other were considered in further analyses (n = 2614). They included 62 call categories, leading to 3,844 possible transitions between call categories but only 289 of these transitions were observed.

**Table 1:** Summary of recordings of Icelandic killer whales (*Orcinus orca*) analysed.

Year	Tag ID	Individual ID	Social cluster	Age-sex class	Sampling rate (kHz)	Recording duration (hh:mm)	No. of calls	No. of transitions	No. of tail slaps	Call combinations (n)
2009	oo09_194a	IS074	L	F	192	04:16	655	250	–	A (198)
	oo09_200a	–	–	–	192	06:28	1751	565	34	C (217)
	oo09_201a	IS071	Q	M	96	04:13	344	93	6	C (2)
	oo09_209a	IS049	D	F	192	01:33	17	6	–	–
2013	oo13_068a	–	–	–	240	00:39	64	7	–	–
	oo13_071a	IS165	B	M	240	01:57	14	–	1	–
	oo13_072a	IS405	L	O	240	02:05	166	26	7	–
2014	oo14_048a	IS011	–	M	192	05:14	28	6	–	–
2021	oo21_175a	IS042	–	M	240	16:06	220	49	1	A (41)
	oo21_182a	IS401	I	M	240	03:18	134	13	1	C (1)
	oo21_183a	IS389	C	J	240	09:46	1267	439	27	A (3) B (263) C (16)
	oo21_184a	IS064	G	F	240	03:17	683	198	–	A (99)
	oo21_186a	IS382	G	O	240	05:57	592	176	30	A (81) C (2)
	oo21_188a	-	–	J	240	00:11	24	8	–	–
	oo21_189a	IS406	L	F	240	01:47	93	22	1	–
	oo21_199a	IS118	L	M	240	01:39	182	40	34	A (7)
	oo21_202b	IS273	–	M	240	02:07	211	66	6	B (35)
2022	oo22_166a	–	–	–	240	04:38	336	92	7	A (69)
	oo22_170a	–	–	J	240	17:10	402	98	16	A(1) C (25)
	oo22_171a	IS266	N	M	240	06:30	472	228	2	A (187)
	oo22_195a*	IS251	–	M	96	10:56	377	146	18	A (67)
	oo22_228a	–	–	J	240	03:01	13	–	2	–
Total						112:48	8045	2528	193	

Recordings were made using Dtags and a CATS Diary tag (*). Individual ID was based on photo-identification from an existing catalogue^54^, and social cluster information was obtained from a previous study conducted on Icelandic killer whales^55^. Age-sex class is given as female (F), male (M), juvenile (J) or other (O) at the time of tagging. The number of calls and tail slaps only includes sounds that were rated as high quality. The number of transitions is the number of call transitions that were within the bout criterion interval. Call combinations refers to clusters of calls that included non-random transitions between specific call categories with the number of transitions in parentheses.

**Figure 1:**
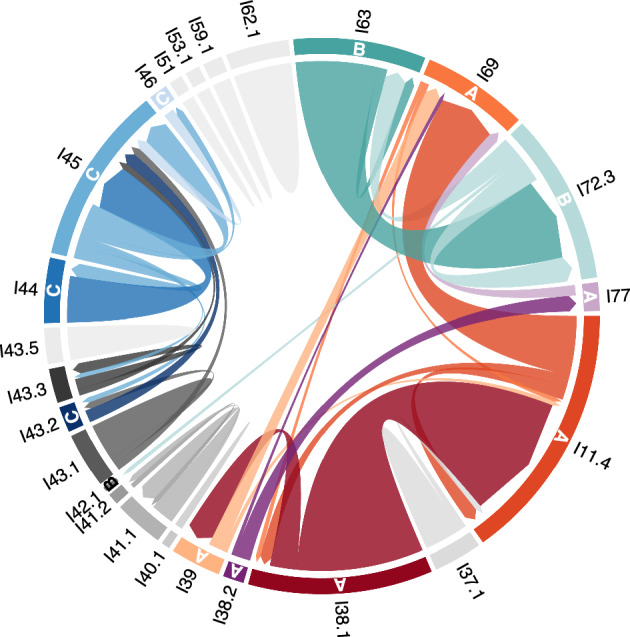
Chord diagram illustrating transitions between call categories of Icelandic killer whales (*Orcinus orca*). The width of each sector in the outer circle represents the sample size. The chords indicate transitions between call categories, with the arrow at the end indicating their direction, and their width reflecting the strength of the relation between call categories (which is proportional to the sample size). Transitions between different categories that occurred significantly more often than expected are highlighted in colours and indicated by letters A, B, and C. Red, orange and purple tones show call combinations of cluster A, turquoise denotes cluster B and blues denote cluster C (see Fig. 2). Other transitions and repetitions of the same call category in greyscale. Transitions that occurred < 10 times were removed for clarity.

**Figure 2:**
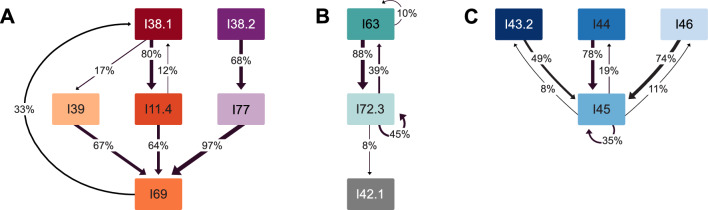
Transitions between different call categories of Icelandic killer whales (*Orcinus orca*). Transitions shown were indicated to be non-random by a Pearson’s chi-square and consequent post-hoc test and occurred > 10 times. Each call category is indicated with a coloured rectangle and transitions are shown with arrows. The thickness and directions of arrows shown represent the probability of one call category being immediately followed by a second category. For example, I38.1 was followed by I11.4 and I39 in 80% and 17% of cases, respectively (and followed by other, not displayed call categories in the remaining 3% of cases). Three distinct clusters of calls were apparent: cluster A consisted of a combination of six different call categories that were given in combinations of two or three, cluster B was a combination of mostly two call categories with infrequent transitions to a third category, cluster C consisted of one call category (I45) that was mainly combined with three other categories.

**Figure 3:**
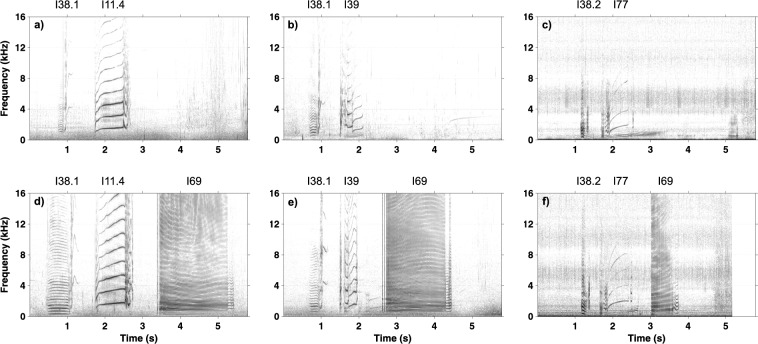
Example spectrograms of call combinations from cluster A recorded from Icelandic killer whales (*Orcinus orca*). Spectrograms (**a**–**c**) show two-call combinations of I38.1 with I11.4, I38.1 with I39, and I38.2 with I77. Spectrograms (**d**–**f**) show three-call combinations with I69 added to each. Recordings sampled at 96 and 192 kHz. Spectrogram parameters: Hann window; 87.5% overlap; FFT size: 4,096; frequency resolution: 23.44 Hz and 46.88 Hz; time resolution: 5.33 ms and 2.67 ms. Corresponding sound files available in Supplementary Audio A1–A6.

**Figure 4:**
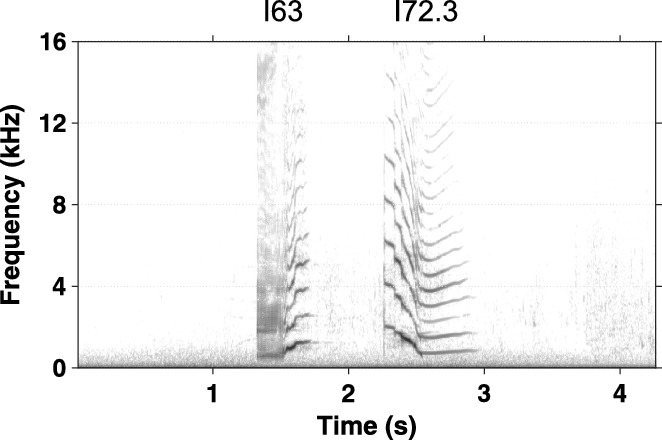
Example spectrogram of the most common call combination from cluster B recorded from Icelandic killer whales (*Orcinus orca*). The combination was of I63 and I72.3 and was often repeated. Recording sampled at 240 kHz. Spectrogram parameters: Hann window; 87.5% overlap; FFT size: 4,096; frequency resolution: 58.62 Hz; time resolution: 2.13 ms. Corresponding sound file available in Supplementary Audio A7.

**Figure 5:**
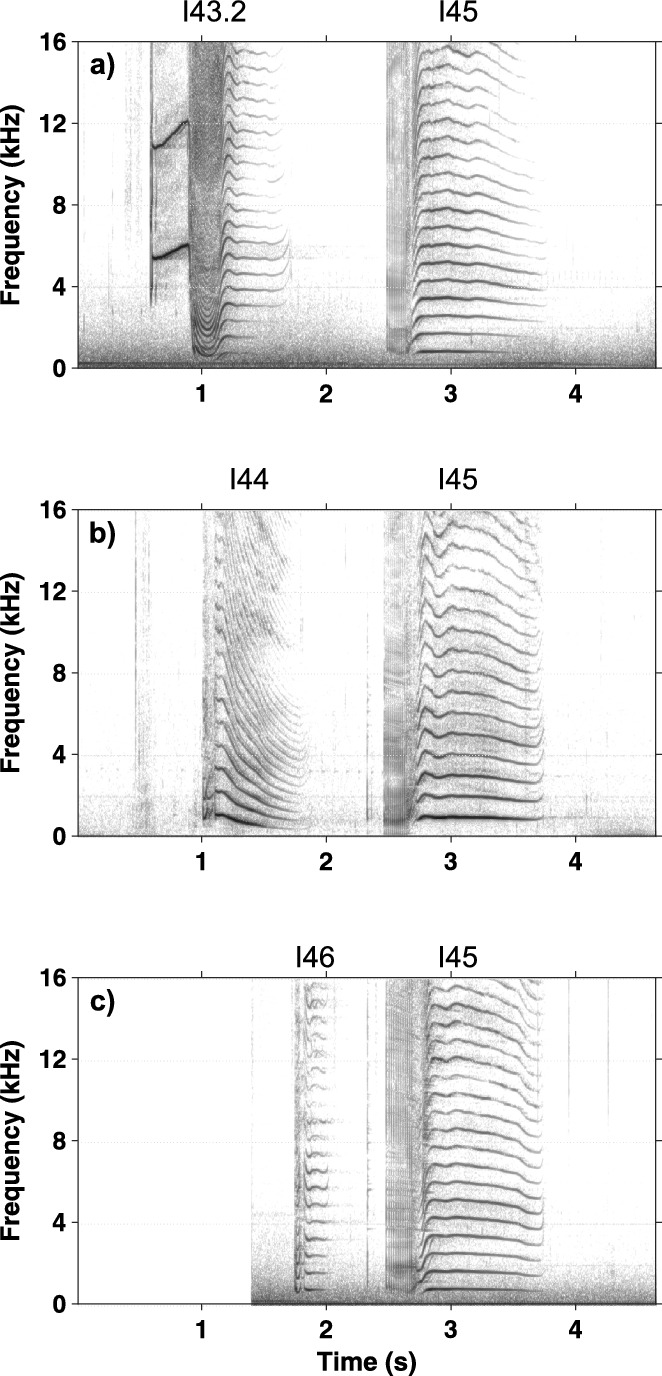
Example spectrograms of the most common call combinations from cluster C recorded from Icelandic killer whales (*Orcinus orca*). In all examples I45 is the second call. It is preceded by (**a**) I43.2, (**b**) I44 and (**c**) I46. Recordings sampled at 192 kHz. Spectrogram parameters: Hann window; 87.5% overlap; FFT size: 4096; frequency resolution: 46.88 Hz; time resolution: 2.67 ms. Corresponding sound files available in Supplementary Audio A8–A10.

First-order Markov chain analysis indicated that transitions between call categories were significantly different from random (χ^2^ = 38,331, p < 0.005). Post-hoc analysis revealed 111 out of the 289 observed transitions that occurred significantly more often than expected. However, many of them (n = 78) occurred only a few times and to avoid influence of these small sample sizes, we illustrate only those that occurred more than 10 times (n = 33, Table 2). Many of these transitions were repetitions of the same call category but specific combinations of transitions across call categories were also observed. Transitions between call categories are illustrated in Fig. 1, showing that specific combinations of call categories were very common. Three distinct clusters of call combinations emerged from the post-hoc analysis, containing transitions that occurred more often than expected by chance. In total, 50.3% of transitions (n = 1314/2614) were part of these call combination clusters and 30.3% of transitions (n = 791/2614) were repetitions of the same call type or same subtype. Most notable was call combination cluster A (n = 753 transitions), consisting of six call categories (I38.1, I38.2, I39, I11.4, I69, I77) that occurred in different combinations of two or three call categories (Figs. 2 and 3, Supplementary Audio A1–A6). The two-call combinations started with I38.1 or I38.2 and mostly transitioned to I11.4 or I39 and I77, respectively. When extended to a three-call combination, the final call was always I69. These combinations were often repeated. The second cluster of calls, cluster B (n = 298 transitions), mostly contained two call categories (I63 and I72.3). Typically, I63 was followed by I72.3 and this combination was often repeated (Figs. 2 and 4, Supplementary Audio A7). The third cluster, cluster C (n = 263 transitions), contained three call categories that were closely linked to I45 (Figs. 2 and 5, Supplementary Audio A8–A10). The consistency in amplitude and the lack of overlap suggested that each call combination was produced by one individual.

**Table 2:**
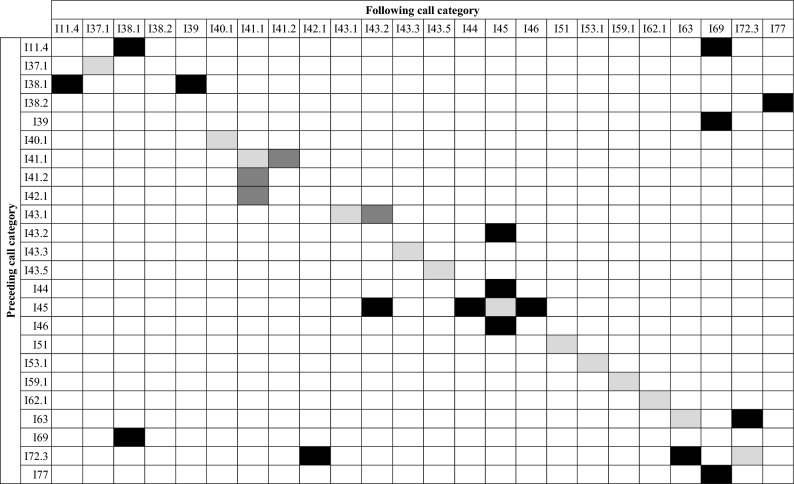
Transitions between call categories of Icelandic killer whales (*Orcinus orca*) that occurred significantly more often than expected based on a Pearson’s chi-square and consequent post-hoc testing (p = 0.05) and that were observed more than 10 times.

Rows are the preceding call category and columns the subsequent call category. Repetitions of the same category in light grey, repetitions of the same call type but different subtypes in dark grey and other transitions marked black. The term ‘call category’ includes both, call types and subtypes (see Methods section).

These call combinations were recorded over several years, across tags on different individuals from different social clusters and from all age-sex classes (Table 1). No call combinations were recorded in 2013 and 2014, which is likely due to small sample sizes in these years (Table 1). Call combinations from clusters A and C were recorded in 2009, 2021 and 2022 and those from cluster B in 2021. Cluster A was recorded on ten tags that were deployed on two individuals without ID and eight different individuals from four social clusters. The combination of I63 and I72.3 (cluster B) was recorded on two tags, on different individuals, one of them from a known social cluster. Combinations of cluster C were recorded on six tags, on four identified individuals, and at least four social clusters.

The majority of call combinations (68.3%, n = 2197/2614 transitions) were produced outside of feeding contexts, i.e. > 5 min from a tail slap. However, this varied greatly between different call combination clusters. Call combinations of cluster C were commonly produced close to tail slaps (78.3%, n = 206/263), while combinations of cluster A (21.7%, n = 163/753) and cluster B (16.1%, n = 48/298) were less commonly produced close in time to tail slaps.

## Discussion

This study shows that Icelandic killer whales rarely produce calls in a random order, instead repetitions and specific call transitions produced within seconds of each other are common. These call combinations were composed of call pairs or triplets and could be assigned to one of three call combination clusters. A total of 50% of the transitions included in the analysis were part of a call combination cluster. They were recorded across a range of tag deployments, over several years, on different individuals from several social clusters and from all age- and sex-classes, indicating that they might play an important role in the communication system of Icelandic killer whales.

A review of acoustic sequences in non-human animals^5^, suggested six paradigms for encoding information in sequences: repetition, diversity, combination, ordering, overlapping, and timing. Our study indicates that at least two of these paradigms (repetition and ordering) are of importance in killer whales. Many call categories were most likely to be followed by themselves, adding to the evidence that repetition of calls is common in killer whales^18,33,34^. The mixed call combinations we describe here, appear to follow an ordering paradigm, where a set of units is combined, and their order is of importance (e.g., AB is different from BA). While all call categories that were part of the mixed call combinations were recorded within and outside of combinations, some (e.g., I38.1 and I69) were found only very rarely outside of combinations. Thus, they might operate like an extension or suffix to other call categories.

Similarly, previous studies on killer whale calls have reported strong associations between call types, with some call types never being produced without the other in the fish-eating resident and mammal-eating transient populations of the North Pacific^32,33,37^. However, it remains unclear whether these repeated and combined units encode information that is different from each individual unit. This can ultimately only be tested using playback experiments, for which a better understanding of the function of different killer whale call types would be essential.

A previous study showed that two lone males of the AT1 transient population in Alaska called in stereotyped patterns, with some three- and four-call sequences given more often than expected^34^. However, unlike in this study, the most common of these sequences were repetitions of the same call types and mixed call combinations made up only a small percentage of the total of calls recorded. In Norway, so-called compound calls were described from killer whales^51,52^. These were defined as multicomponent calls consisting of more than one discrete call. They were differentiated from other call types with multiple components in that all calls composing the compound call had to also be given individually as discrete calls or they had to be used in combinations with different discrete call types^51,52^. Most of these multicomponent calls were near continuous concatenations of sounds. The call combinations from Icelandic killer whales described here consist of individual sounds that were clearly separated by a period of silence shorter than the BCI of 1.72 s.

Most striking were call combinations derived from cluster A, which included six call categories that were given in sequences of two or three. The combinations were highly stereotyped and, although different combinations were generated, there were clear rules regarding which were the first and last call types used. This makes these combinations very conspicuous in acoustic recordings and they are even picked up on by novice listeners. Call combinations from this cluster were found in ten out of 22 tags, which were deployed over a 13-year period and placed on individuals from four social clusters, plus two individuals of unknown social clusters and two unknown individuals. While all these tags were deployed in Vestmannaeyjar (South Iceland), call combinations from cluster A were also noticed in other recordings, including from a moored hydrophone deployed in Breiðafjörður (West Iceland) in March 2014, as well as from single hydrophones and towed arrays in Vestmannaeyjar in July 2015 and 2016. This indicates that these call combinations are widely spread and commonly used.

The function of call combinations in Icelandic killer whales remains unclear. In other species, vocal sequences have been related to a variety of contexts, including predation^56^, travelling^10,11,57^, social^58^ or feeding contexts^26,27,59,60^. Most individual killer whale calls do not seem to be behaviour specific^33,47^, leading to the suggestion that it could be the combination of sounds rather than the individual calls that are of importance^46^. While call combinations comprised only part of the killer whale vocal production, they were produced in most tags, on individuals from different social clusters and all age- and sex-classes, as well as in most years sampled. In some tags call combinations composed a large part of the call production (Table 1), indicating that these combinations may be important during specific behaviours. Our results indicate that call combinations of cluster C may be related to feeding, but those of clusters A and B mostly occurred outside of feeding contexts. Icelandic killer whales are most vocal during feeding, followed by socialising, and almost silent when travelling^61^. The social complexity hypothesis suggests that social contexts may place additional demands on the communication system, favouring the combination of calls^2^. Since call combinations of clusters A and B were common but occurred mostly outside of feeding contexts, they could indicate socialising. However, we only provide a basic and preliminary investigation into behavioural context based on the acoustic record of the tags and only distinguish feeding and non-feeding contexts. Detailed studies using the other tag sensor data (e.g. accelerometer, magnetometer, depth, video), that were outside the scope of this study, could provide more comprehensive information on this topic in the future.

Previous studies on killer whale and narwhal vocal sequences suggested that they may play a role in long-distance communication with other group members^31,34^. Recent work on killer whale sounds shows that as ambient noise levels increase, high frequency components of killer whale calls and buzzes with energy peaks in higher frequencies may be detectable over longer distances^62^. While none of the call categories in the described combinations contained a second, high frequency component, all combinations contained calls with buzz components. In particular, I69 (call combination cluster A) is a long, buzz-like call. Such a sound should transmit over long distances and contains frequencies that lie within the best hearing range of killer whales^62^. The spectrograms presented in the study^62^ also indicate that while individual calls might be rendered unrecognisable with distance or increasing noise, the structure of a sequence of calls could be retained and contribute to detectability over long distances. Therefore, call combinations and in particular those of cluster A could be used in long distance communication but further study, e.g. on source levels, would be required to determine their function.

Another possibility is that call combinations serve as identifiers of different individuals or social groups, as has been suggested for long-finned pilot whales^20^. Icelandic killer whales live in a fission–fusion society, where associations between individuals are non-random and some strong social bonds exist, allowing for a grouping of individuals into social clusters^55^. Due to the strict quality criteria applied in this study, we consider it likely that the calls described herein were either produced by the tagged whale itself or another individual in very close proximity, which would likely belong to the same social cluster. While it is unclear whether Icelandic killer whales have group-specific repertoires, there is variation in the repertoire with location^44^ and differences in call combination patterns between social clusters would further support the idea of some differentiation in calling behaviour between groups. While none of the call combination clusters appeared to be exclusive to a single social cluster, several social clusters only produced one of the three call combination clusters. Thus, we have little evidence of whether or not call combinations could be group specific. So far, we only covered nine of the 18 described social clusters^55^ and further group-specific recordings are required to clarify whether call combinations could be group markers.

This study provides a first detailed description of call combination patterns, not likely to occur by chance, produced by Icelandic killer whales. Some of these sequence patterns are, at least in part, similar to what has been described from other populations, but lack of consistency in methodology and definitions used hinders further comparisons. A cross-population comparison of call combinations using a unified approach could be highly informative for the study of killer whale acoustic behaviour and call evolution. Furthermore, linking the detected call combinations to specific behaviours could provide information on the function of these combinations which would be beneficial e.g., for the interpretation of long-term passive acoustics data. For the Icelandic population in particular, group-specific recordings and further investigation of their social structure is required to better understand the usage of call combinations.

## Methods

### Data collection

Data were collected in 2009 and 2021–2022 in Vestmannaeyjar, South Iceland, and in 2013–2014 in Breiðafjörður, West Iceland. These locations are herring spawning and overwintering grounds, respectively, and killer whales are known to gather there in large numbers during summer or winter/spring to feed on herring^63^. Digital acoustic recording tags (Dtags, flat frequency response: 0.6–45 kHz)^64^ and a Customized Animal Tracking Solutions (CATS; www.cats.is) Diary tag with an integrated hydrophone (HTI-96-Min; flat frequency response up to 30 kHz) were attached to killer whales using a carbon fibre pole or pneumatic tag launcher (ARTS)^65^. Tags attached to the body of the whale with suction cups. During some tag deployments, animals were subject to playback experiments. Only data prior to the start of sound transmission were used from those deployments.

### Acoustic analysis

Acoustic recordings from the tags deployed in 2013–2022 were inspected using spectrograms (Hann window, NFFT = 8,192) in the software Audacity version 2.3.1 (www.audacityteam.org). For the tags deployed in 2009, the software Adobe Audition 2.0 (Adobe Systems Inc., San Jose CA) was used to generate the spectrograms (Blackmann-Harris window, FFT = 2048 or 4096, for 96 and 192 kHz sampling rates, respectively). Calls were given a quality rating of high, medium, or low based on the perceived signal to noise ratio (SNR) and overlap with other sounds. Only high-quality calls were extracted. These calls were clearly visible and audible, showed several sidebands, and had little or no overlap with other sounds. The SNR was measured whenever possible using a custom routine in MATLAB version 9.14.0 (R2023a, www.mathworks.com). The routine compared the sound pressure level of the call to that of a 200 ms segment of ambient noise (without any calls or other transients) within a few seconds before the call. The sound pressure level of the call was based on its 90% sound energy duration. Recordings were filtered using a 3rd-order Butterworth bandpass filter with cut-off frequencies of 450 Hz and 10 kHz to retain the dominant frequency range of Icelandic killer whale calls^66^. Generally, only calls with a SNR higher than 10 dB were used in further analyses. However, in a few cases (n = 235, 3%) flow or boat noise around and below 1 kHz appeared to influence the SNR measurements while contours of the call were clearly visible at higher frequencies. These calls were included despite a SNR below 10 dB. Calls from 2009 to 2014 were previously classified to call type and subtype and included in a call catalogue for Icelandic killer whales^44,67^. This classification was validated using classification and regression tree (CART) and random forest analyses, as well as an interobserver test with 11 observers^44^. Calls from 2021–2022 were classified by AS following the same protocol as used in the call catalogue^44^. We use the term ‘call category’ to include both, call types and subtypes. New call categories were created whenever appropriate, following the same protocol. Variable calls that did not appear to be stereotyped were marked as ‘unknown’.

### Sequence analysis

Preliminary inspection of the recordings showed that possible combinations of calls occurred with relatively short silent gaps of around 0.5–1.0 s. Like many other behaviours, calling occurs in bouts and a bout criterion interval (BCI) can be determined to separate behaviours within and between bouts. The BCI can be identified based on the gap between behaviours, where the distributions of gaps are considered to be a combination of two or more Poisson processes that separate behaviours within bouts (fast process) from behaviours between bouts (slow process)^68^. In acoustic behaviours, the behavioural gap is the silent interval between sounds, which was calculated by subtracting the end time of a call from the start time of the following call. The BCI was determined based on maximum likelihood estimation^69,70^, using the fitMLEbouts function of the DiveMove package^71^ in R version 4.1.2 (www.R-project.org). Based on exploratory analysis, only pauses less than 30 s were included to reduce potential bias introduced by a few very long pauses.

Transitions between call categories that were within the BCI were analysed using a first-order Markov model. Expected and observed transition matrices were compared using a Pearson’s chi-squared test to test the null hypothesis that transitions were random. As several of the expected occurrences of transitions were low, a Monte Carlo simulation with 2000 replicates was applied to calculate the p-values (stats package in R). A post-hoc test for pairwise comparisons was run using the ‘chisq.posthoc.test’ package^72^ with a Bonferroni correction for multiple testing. Multiple category sequences were specified to have occurred when all first-order transitions in the sequence were found to be significantly unlikely due to chance, in the post-hoc analysis.

### Individual identification and behavioural context

Photographs for identification of individual whales were taken during tagging and/or when the tag was on the whale. Individuals were identified from the shape and size of the dorsal fin, the saddle patch, and nicks and scars present on the body^73^, and their photographs were matched to an existing photo-identification catalogue^54^. Age- and sex-class were assigned either based on body size and the shape and size of the dorsal fin, or based on genetic sexing. Males were defined as adult or sub-adult individuals with a distinctly taller dorsal fin, females as mature in size and consistently seen with a calf in echelon position, and juveniles as more than one year of age but not mature-sized. Other mature-sized individuals without clear sex determination were marked as ‘other’^63^. Social cluster information was obtained from a previous study on social structure in Icelandic killer whales^55^.

Acoustic records of the tags were inspected to investigate whether call combinations were related to feeding contexts. Killer whales feeding on herring employ a carousel feeding strategy, in which they use tail slaps to debilitate herring^74^. The tail slaps are clearly audible in nearby acoustic recordings^75^. The acoustic record of each tag was inspected for tail slaps and each was given a quality rating of high, medium, or low. Only high-quality tail slaps were included in the analysis to ensure that the tail slaps were produced either by the tagged whale or an individual in close proximity. Call combinations occurring within five minutes of a tail slap were considered to be produced in a feeding context, as this time interval captured feeding contexts well in previous studies^76^.

### Supplementary Information

Supplementary Information 1.

Supplementary Information 2.

## Data Availability

Data used in this study (comprising a list of calls used in this study, their start/end times, pauses between calls, and signal to noise ratio) is available in the Supplementary Information.
